# Thermoelectric Transport in a Three-Dimensional HgTe Topological Insulator

**DOI:** 10.3390/nano11123364

**Published:** 2021-12-11

**Authors:** Gennady M. Gusev, Ze D. Kvon, Alexander D. Levin, Nikolay N. Mikhailov

**Affiliations:** 1 Instituto de Física, Universidade de São Paulo, São Paulo 135960-170, Brazil; alevin@if.usp.br; 2 Institute of Semiconductor Physics, 630090 Novosibirsk, Russia; kvon@isp.nsc.ru (Z.D.K.); mikhailov@isp.nsc.ru (N.N.M.); 3 Novosibirsk State University, 630090 Novosibirsk, Russia

**Keywords:** topological insulator, thermopower, quantum transport, HgTe quantum well

## Abstract

The thermoelectric response of 80 nm-thick strained HgTe films of a three-dimensional topological insulator (3D TI) has been studied experimentally. An ambipolar thermopower is observed where the Fermi energy moves from conducting to the valence bulk band. The comparison between theory and experiment shows that the thermopower is mostly due to the phonon drag contribution. In the region where the 2D Dirac electrons coexist with bulk hole states, the Seebeck coefficient is modified due to 2D electron–3D hole scattering.

## 1. Introduction

A three-dimensional topological insulator (3D TI) has a gapless surface state inside the bulk band-gap [1,2,3,4]. The surface state energy spectrum has the form of a Dirac cone, which holds massless particles. Remarkably, the spin of surface Dirac electrons is locked perpendicular to the wave vector *k* in the 2D plane, which leads to the suppression of the electron scattering on impurities. The wide strain HgTe films are among of the best host 3DTI materials [5,6] because, in such a system, a very high mobility of 2D surface electrons μ~100 m^2^/V·s is achieved [7,8,9,10].

The thermoelectric measurements can probe the sign of the charge carriers and the transport mechanisms and are widely used to obtain complementary information about electron transport in metals and semiconductors. Moreover, the value of the thermoelectric coefficient strongly depends on the energy spectrum and the mechanism of the time relaxation. For example, an important relationship exists between the diffusive thermopower $S_{xx}$ and the logarithmic derivative of the longitudinal electric conductivity $\sigma_{xx}$ of a metal:

(1) $$S_{xx}=-\frac{\pi^{2}}{3e}k_{B}T\frac{d}{d\mu }\left[ln\sigma_{xx}\left(\mu \right)\right]$$

where *μ* is the chemical potential of charge carriers. Based on the Mott relation, anomalously large thermopower has been predicted in a 2D topological insulator [11]. A 2D TI is characterized by a pair of counterpropagating gapless edge modes inside of the bulk gap [12]. These edge states have helical spin properties and are proposed to be robust to backscattering [1,3,13]. It is suggested [11] that when the Fermi level approaches the conduction or valence band edge, the scattering rate of electrons in the helical one-dimensional modes increases significantly due to 1D–2D scattering, which leads to the anomalous growth of the amplitude of the Seebeck signal and a change of its sign. However, note that this mechanism requires the complete suppression of the scattering between the edge states, which is not observed in realistic structures [13]. An experimental probe of the thermoelectric response demonstrates that the observed thermopower is mostly due to the bulk contribution, while the resistance is determined by both the edge and bulk transport [14].

A similar situation is expected in a 3D TI: when the Fermi level crosses the edge of the bulk bands, additional 2D–3D scattering can lead to an increase in thermopower coefficients. Such mutual scattering has been detected directly in the resistance behavior [8,9]. Note that recently, 2D electron–3D hole scattering has been deduced from the nonmonotonic differential resistance of narrow 3DTI HgTe channels [15].

Another system, where the coexistence of the two distinct types of carriers with a different charge sign affects the transport properties, is a 2D semimetallic HgTe well of intermediate well width (≈20 nm) [16,17]. In this system, 2D electron–2D hole scattering directly results in temperature-dependent resistivity ρ, which increases with temperature as $\rho \sim T^{2}$ in accordance with the prediction for electron–hole friction coefficient behavior [18]. The thermopower in such a system has been studied in papers [19,20]. A comparison between theory and experiment demonstrated that the observed thermopower in a 2D electron–hole system is mostly due to phonon drag. It has been argued that the role of 2D electron–2D hole scattering is important in the formation of thermoelectric power mechanisms.

Thus, thermoelectric power is a very important tool to study the mechanism of scattering between carriers of different signs and even between carriers of different dimensions, as for example 1D–2D (2D topological insulators [11]) and 2D electron–2D holes (2D semimetals [19]).

In the present paper, we report an experimental study of the thermoelectric response in 80 nm thick strained HgTe layers. We found that thermopower in a 3DTI is due to phonon drag, which is similar to a 2D semimetal system in 20 nm HgTe wells. When the Fermi level crosses the region with coexistence of 2D electrons and 3D holes, mutual scattering causes a strong change in thermopower.

## 2. Materials and Methods

**Figure 1:**
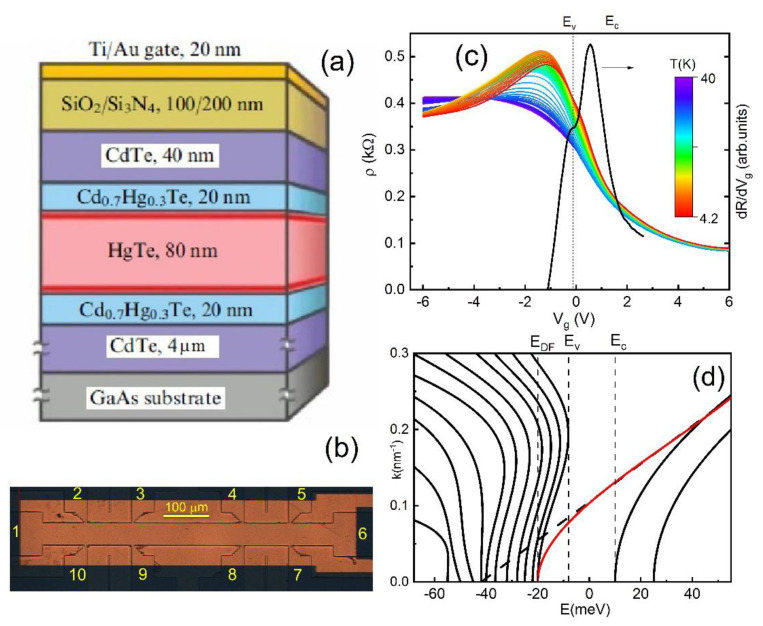
(**a**) Schematic of the transistor and (**b**) Top view of the sample. (**c**) Resistivity ρ as a function of gate voltage measured for different temperatures. The derivative of the resistance *dR/dV_g_* as a function of gate voltage at *T* = 4.2 K. (**d**) Schematic of the energy spectrum of a strained 80 nm mercury telluride film. Conduction and valence band edges are marked by $E_{c}$ and $E_{v}$, the edge band of the surface states (the Dirac point), which is located in the valence band, by $E_{DF}$. Dashes represent the spectrum of interface states under approximation where the mixing of these states with bulk hole states is neglected.

**Figure 2:**
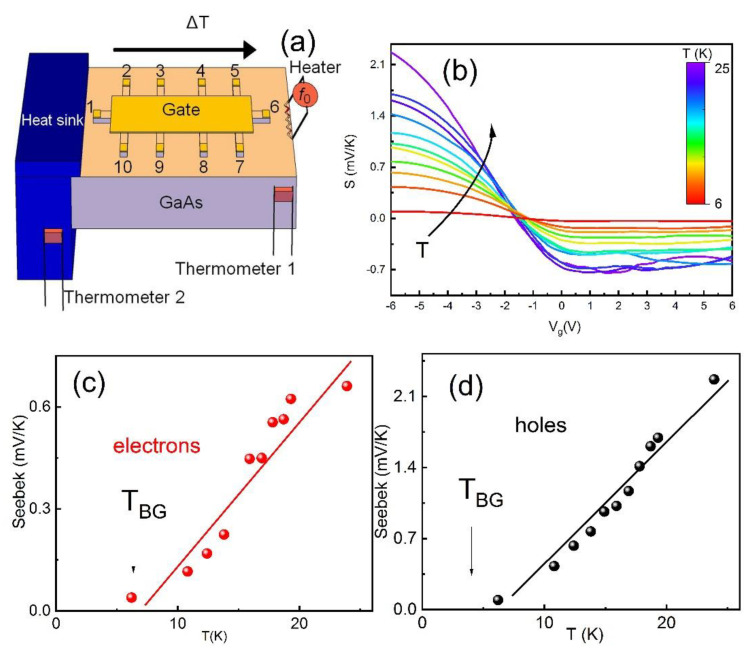
(**a**) Sample geometry. (**b**) Seebeck coefficient as a function of the gate voltage for different temperatures. (**c**) Temperature dependence of Seebeck coefficient at $V_{g}=6\;V$ (electrons). (**d**) Temperature dependence of Seebeck coefficient at $V_{g}=-6\;V$ (holes). The solid lines correspond to $S_{xx}\sim T$. Arrows indicate Bloch–Gruneisen temperature.

The HgTe material was grown by molecular beam epitaxy on (013)-oriented GaAs. The sample was an 80 nm HgTe layer that is sandwiched between two Hg_0.3_Cd_0.7_Te buffer layers above and below (40 nm) (Figure 1a). The details of the structural properties of the prepared sample have been published in a previous paper [21]. For transport measurements, a field effect transistor was used. The sample was a long Hall bar consisting of three 50 μm wide consecutive segments of different length (100, 250, and 100 μm) and eight voltage probes (Figure 1b). The top of the Hall bar device was covered by a dielectric layer and subsequently a metallic gate. A pyrolytic SiO_2_ layer or a double SiO_2_ + Si_3_N_4_ layer grown at temperatures of 80–100 °C was used as a dielectric, and the TiAu layer served as a gate. The resistance measurements were performed in a variable temperature insert cryostat in the temperature range 1.4–50 K using the standard four-point scheme. The electrically powered heater was glued symmetrically near Contact 1 (see Figure 2a) and created a temperature gradient in the system, while the other end was indium soldered to a small copper slab that served as a thermal ground. One calibrated thermo sensor was attached at the end of the sample near the heater, while the other was attached to the heat sink (see Figure 2a). Thermo sensors were used to measure the ΔT along the sample. For typical heat power, we applied ∇T=160 K/m. The voltages induced by this gradient were measured by a lock-in detector at the double frequency of $2\;f_{0}=0.8÷2\;\mathrm{Hz}$ across various voltage probes. The thermovoltage was proportional to the applied power. The thermal conductance of the sample was overwhelmingly dominated by phonon transport in the GaAs substrate [14]. Three samples were measured.

## 3. Results

Figure 1c presents the resistances at zero magnetic field for the sample fabricated from an 80 nm HgTe layer for different temperatures. The current I flows between Contacts 1 and 6, and the voltage V is measured between the short distance separated Probes 2 and 3, $R=R_{1,6}^{2,3}$ (Figure 1b). The temperature dependence $R\left(V_{g},\;T\right)$ reveals the different character of the transport in the different regions of the energy spectrum, and it has been performed in the previous publications [8,9]. Figure 1d shows the specific features in the energy spectrum of the strained HgTe layer. It is worth noting that the lattice constant of CdTe is slightly larger than that of HgTe, inducing strain on the sample. The strain opens an energy gap in the energy spectrum. The Dirac point in the 3D TI ($E_{DF}$) is located deep in the valence band, and due to hybridization with the valence band, the spectrum of the surface states, consisting of Dirac electrons near the bottom of the valence band, deviates from a linear law (Figure 1d, red line). Thus, when the gate voltage is swept from negative to positive values, the electrochemical potential μ moves from the conductance band ($\mu >E_{c}$), through the bulk gap ($E_{v}<\mu <E_{c}$) to the valence band ($\mu <E_{v}$), as one can see in Figure 1d. However, note that the energy scale in Figure 1d does not necessarily correspond with the gate voltage scale in Figure 1c due to the nonmonotonic behavior of Fermi energy with density. For example, the point $E_{DF}$ corresponds to the gate voltage $V_{g}=-20\;V$ because of the high density of the states in the valence band. Since dielectrics breakdown may occur at lower gate voltages, we cannot approach this energy point. Resistance in the bulk gap region originates from the helical surface electron states, with different densities in the top and the bottom surface [8]. In the region $E_{DF}<\mu <E_{v}$, two-dimensional surface electrons and 3D bulk holes coexist. Nonmonotonic temperature dependence of the resistance is observed: R(T) increases for temperatures below 15 K while decreasing above 20 K. We attribute this behavior to 2D electron–3D holes scattering, which is similar to 2D electron–2D hole scattering in HgTe semimetal wells [17,18]. Figure 1d shows that the derivative of resistance $dR/dV_{g}$ reveals the features in points $E_{c}$ and $E_{v}$, correspondingly. In the region $\mu <E_{DF}$, the transport is determined by 3D bulk holes, and we do not expect peculiarities in resistance and thermopower behavior. The position of the energy $E_{DF}$ can be obtained approximately from a detailed analysis of the Shubnikov de Haas oscillations [7].

Once we have determined the gate voltage and the density interval with different transport properties, we now turn to study the thermoelectric response in our films. Experimentally measured quantities were the longitudinal thermovoltage $V_{xx}=S_{xx}\nabla TL$, where L=450 μm is the distance between Probes 1 and 6 along the temperature gradient ΔT (Figure 2a). We also measured longitudinal thermovoltage between Probes 2–3 and 2–5 in order to examine the homogeneity of the temperature gradient, and we found reasonable proportionality to L. Particularly, we found the ratio of the signal *V_1,6_/V_2,3_ =* 6–8, and *V_1,6_/V_2,3_ =* 1.5–2, which approximately agrees with the distance between probes.

Figure 2b shows the gate voltage dependencies of the Seebeck coefficient $S_{xx}$ at different temperatures in the zero magnetic field. The thermopower is negative in the region $E_{v}<\mu$. Upon a further decrease in the gate voltage toward hole contribution, the Seebeck coefficient changes the sign, crossing the zero at the voltage corresponding to the transition from electron-dominant to hole-dominant contribution, which is coincident neither with the charge neutrality point, determined from the zero Hall resistance measurement in a magnetic field ([9,10]), nor with positions of energies $E_{v}$ or $E_{DF}$. The value of the Seebeck coefficient is larger for the holes. Figure 2b displays the traces of $S_{xx}$ versus $V_{g}$ for different temperatures. Figure 2c,d show the temperature dependence of the Seebeck coefficient module measured at selected gate voltages $V_{g}=6V$ and −6 V, corresponding to the electron and hole-dominated regions. One can see that $S_{xx}$ grows linearly with temperature as $S_{xx}\sim \;T$ in the interval 10<T<25 K. In the region $E_{DF}<\mu <E_{v}$, where 2D electrons and 3D bulk holes coexist, we are not able to distinguish the thermopower mechanism due to electron–hole scattering, which can modify the temperature dependence of $S_{xx}\left(T\right)$, and for which a comparison between the theory and the experiment requires more advanced theory. We should note that previous research in 3DTI does not reveal semimetallic behavior and features in the resistivity and thermopower due to e-h frictions, since samples have a lower mobility [22].

## 4. Discussion

Detailed calculations of thermopower in 2D semimetals have been performed in papers [19,20], where both contributions (diffusion and phonon drag) have been taken into account. The Seebeck coefficient in the zero magnetic field is given by:

(2) $$S_{xx}=\frac{\Lambda_{x}}{Z}$$

(3) $$Z={\left[m_{e}n_{h}\tau_{h}+m_{h}n_{e}\tau_{e}+{\left(n_{e}-n_{h}\right)}^{2}\eta \tau_{e}\tau_{h}\right]}^{2}$$

(4) $$\Lambda_{x}=\frac{\sqrt{Z}}{e}\left[A^{e}\tau_{e}m_{h}-A^{h}\tau_{h}m_{e}+\left(A^{e}+A^{h}\right)\left(n_{e}-n_{h}\right)\eta \tau_{e}\tau_{h}\right]$$

where $A^{e,h}=A_{dif}^{e,h}+A_{ph-dr}^{e,h}$ are the electron and hole terms corresponding to the diffusion and phonon drag contributions to the Seebeck coefficient respectively, $n_{e}$ and $n_{h}$ are the electron and hole densities, $m_{e}=0.03\;m_{0}$ and $m_{h}=0.3\;m_{0}$ are the electron and hole effective mass, $g_{e}=1$ and $g_{h}=2$ are the electron and hole valley degeneracy, $\tau_{e}$ and $\tau_{h}$ are the electron and hole transport scattering time, determined from the electron and hole mobilities respectively, k is the Boltzmann constant, and $\eta =\Theta \times T^{2}$ is the electron–hole friction coefficient. Parameter Θ is introduced in paper [17] for scattering between 2D electrons and 2D holes. It is determined by the peculiarities of the interaction between electrons and holes and dependents of their densities. The diffusion contribution does not contain any adjustable parameters and is given by

(5) $$A_{dif}^{e,h}=-\frac{\pi }{3\hbar^{2}}k^{2}Tm_{e,h}g_{e,h}.$$

The phonon drag contribution depends on the material specific phonon relaxation rate and the temperature regime. The system enters into the Bloch–Gruneisen (BG) regime at a very low temperature T when the acoustic phonon wave vector $q=2k_{F}$, where $k_{F}$ is the Fermi wave vector. In our HgTe system, we found that for the densities $n_{s}\sim 10^{12}{\mathrm{cm}}^{-2}$, the characteristic temperature $T_{BG}=\frac{2k_{F}s\hbar }{k}$ (s is the sound velocity) is around 4−6 K (Figure 2c,d). If we propose that $\tau_{ph}=const$, then for temperatures $T\gg T_{BG}$, we obtain:

(6) $$A_{ph-dr}^{e,h}=-\frac{1}{3\hbar^{5}}k\tau_{ph}m_{e,h}^{2}sp_{F_{e,h}}^{2}g_{e,h}B_{e,h}\left(q_{T}\right),$$

where $q_{T}=kT/\hbar s$ is the thermal phonon wave vector, and for acoustic phonons in cubic crystals, the function $B_{e,h}\left(q_{T}\right)$ reads:

(7) $$B_{e,h}\left(q_{T}\right)=\frac{\Lambda_{e,h}^{2}q_{T}}{2\delta s\;}$$

where $\Lambda_{e,h}$ are the deformation potential constants, and δ is the crystal density. The data for the deformation potential: $\Lambda_{e}=-4.8\;\mathrm{eV}$ and $\Lambda_{e}=-0.92\;\mathrm{eV}$; for δ and s, we have: $\delta =8.2\;g/{\mathrm{cm}}^{3}$, $s=3.2\times 10^{5}\mathrm{cm}/s$. One can see that in the temperature regime $T\gg T_{BG}$, both contributions $A_{dif}^{e,h}$ and $A_{ph-dr}^{e,h}$ linearly depend on temperature.

Note that in the monopolar limit in the regions $\mu <E_{DF}$, where the transport is determined by 3D bulk holes, we obtain the following equations for diffusive and drag contributions (using subscript h):

(8) $$S_{dif,ph-dr}^{e,h}=\pm \frac{A_{diff,ph-dr}^{e,h}}{en_{e,h}}.$$

In the conduction band in the region $\mu >E_{c}$, the bulk and surface carriers coexist; however, for simplicity, we applied Equation (8) (using subscript n), where $n_{e}$ is the total bulk and surface densities. For simplicity, we consider the situation when the top and bottom surfaces are equally occupied. Since our HgTe layer is not thick, bulk carriers can be considered as a quasi-two-dimensional system (see Figure 1d).

**Figure 3:**
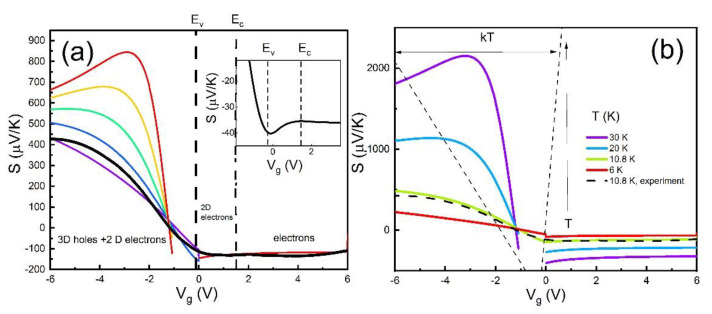
(**a**) Seebeck coefficient as a function of the gate voltage calculated for Equations (2)–(8) with parameters indicated in the text and for different parameter Θ values: $0.1,\;0.25,\;0.5,\;1,\;2,\;4\;(\times 4.8\times 10^{-38}\;J\cdot s/K^{2}$). The black line is the Seebek coefficient measured at T=10.8 K. The insert shows the $V_{g}$ dependence of the Seebeck coefficient zoomed-in on the voltage interval $E_{v}<\mu <E_{c}$ at T=4.2 K. (**b**) Seebeck coefficient as a function of the gate voltage calculated for Equations (2)–(8) with parameters indicated in the text and for different temperatures. Horizontal arrows show the interval where μ~kT, and where degenerate approximation for electrons and holes is not valid any more. Therefore, Equations (2)–(8) cannot be applied to this region. A dashed line corresponds to the measured Seebeck coefficient at 10.8 K.

Figure 3a shows the comparison between the theoretical Seebeck coefficients calculated according to Equations (2)–(8) for different parameter Θ values and the experimental curve measured at T=6 K as a function of $V_{g}$. Parameter Θ is associated with the electron–hole friction coefficient η, and it is responsible for features in thermopower in the region $E_{DF}<\mu <E_{c}$, where surface electrons and bulk holes coexist. Figure 3b shows the comparison between the theoretical Seebeck coefficients calculated according to Equations (2)–(8) for different temperatures as a function of $V_{g}$. We obtain good quantitative and qualitative agreement with the theory.

The diffusion contributions in the monopolar regions $S_{dif}^{e,h}$ were calculated according to Equation (5) for the sample parameters, determined from conductivity. No adjustable parameters have been used in the calculation of the diffusive thermo emf. The phonon drag contribution in the monopolar regions $S_{ph-dr}^{e,h}$ was calculated according to Equations (6)–(8), using a constant phonon relaxation time $\tau_{ph}=0.6\times 10^{-7}s$, corresponding to the relaxation length $l_{ph}=s\tau_{ph}=0.2\;\mathrm{mm}$, which is close to the sample size. We argue that the phonon relaxation length is determined by their scattering on the substrate boundaries. Since the diffusion contribution was calculated without adjustable parameters, we found that $S_{dif}^{e,h}\ll S_{ph-dr}^{e,h}$; therefore, one may conclude here that the phonon drag contribution is the dominant contribution at high temperature T>4.2 K. As we already mentioned above, the temperature dependence of Seebeck coefficient is linear for temperature above $T_{BG}$ for both contributions.

In the bipolar region $E_{DF}<\mu <E_{c}$, where surface electrons and bulk holes coexist, thermopower was calculated according to Equations (2)–(7). We used a simplified model, assuming that the electron–hole friction coefficient η does not depend on the electron and hole densities. Indeed, this model is much too simple to adequately describe the shape of thermopower behavior in this region. Figure 3a demonstrates that the shape of the curve $S\left(V_{g}\right)$ is closer to the experimental one for $\Theta =1.0\times 10^{-38}\;J/K^{2}$, which is smaller than the friction coefficient for the 2D electron and 2D hole system $\Theta =4.8\times 10^{-38}\;J/K^{2}$ [18]. The insert shows the Seebeck coefficient zoomed-in on the voltage interval $E_{v}<\mu <E_{c}$ for T=4.2 K. One can see that thermopower is enhanced near point $\mu ≅E_{c}$, which we attribute to the 2D Dirac electron–3D bulk holes scattering. The feature near $E_{v}$ is smeared out at higher temperatures. Note that the equations in the monopolar regime (8) cannot be obtained from Equations (3) and (4) through a transition to the monopolar case. It is because they are obtained under the assumption that Fermi gases are degenerate. Indeed, the transition to the monopolar limit at low temperatures occurs in a relatively narrow range of the chemical potential Δμ~T (see Figure 3b). It may lead to discontinuity in the calculated thermopower around transition points. While our experiment offers an interesting outlook on thermopower in this region, more experimental and theoretical work is required to understand the behavior of the friction between 2D electron and 3D holes in a 3D topological insulator.

It is worth noting that we also compared the experiment with the monopolar model (Equation (8)), considering independent 2D electron and 3D hole contribution to thermopower only (not shown here). Indeed, we obtained considerable disagreement between the theory and the experiment, which supports the evidence of mutual electron–hole friction in our system [7,8,15].

Figure 3b shows the temperature dependence of the theoretical curves $S\left(V_{g}\right)$. As we expected, in the monopolar region, $S\left(V_{g}\right)$ is proportional to the temperature in accordance with experimental observations (Figure 2c,d), while in the bipolar region $E_{DF}<\mu <E_{c}$, the Seebeck coefficient $S\left(V_{g}\right)$ grows with temperature faster due to mutual friction temperature dependence $\eta =\Theta \times T^{2}$. We do not see such behavior in the experiment (Figure 2b). As we mentioned above, the model is valid for degenerate Fermi gases and cannot be applied at high temperatures near the charge neutrality point. More advanced theory is required to describe this behavior, which is out of the scope of our experimental paper.

## 5. Conclusions

In conclusion, this work is the first to study the behavior of the thermo emf of a three-dimensional topological insulator based on an HgTe 80 nm thick film. The obtained experimental dependencies are compared with a theory. When the gate voltage is sweeping from negative to positive values, the electrochemical potential μ moves from the conductance band ($\mu >E_{c}$) through the bulk gap ($E_{v}<\mu <E_{c}$) to the valence band ($\mu <E_{v}$), and we expect a different thermopower regime. In the monopolar regimes, we demonstrate the calculated values of the transport coefficients corresponding to the drag contribution, which is approximately an order of magnitude larger than the diffusion thermopower. Taking this contribution into account, we determine the phonon relaxation length, which turns out to be temperature independent and caused by phonon scattering at the structure boundaries. In the bipolar region $E_{DF}<\mu <E_{c}$, where surface electrons and bulk holes coexist, the Seebeck coefficient is modified due to 2D electron–3D hole scattering. Comparison with the theory demonstrates good agreement; however, exact knowledge of the mutual friction behavior is required for a better understanding of thermopower in such a nontrivial regime.

## Data Availability

The data presented in this study are available on request from the corresponding author.
